# Efficacy of turmeric in the treatment of oral mucositis in patients with head and neck cancer after radiotherapy or chemoradiotherapy: a systematic review and meta-analysis

**DOI:** 10.3389/fphar.2024.1363202

**Published:** 2024-03-15

**Authors:** Chiu-Feng Wu, Hui-Juan Wu, Chia-Lung Shih, Tzu-Pei Yeh, Wei-Fen Ma

**Affiliations:** ^1^ Department of Nursing, Ditmanson Medical Foundation Chia-Yi Christian Hospital, Chiayi City, Taiwan; ^2^ Doctoral Candidate, Department of Public Health, China Medical University, Taichung, Taiwan; ^3^ Institute of Medical Informatics, National Chung Cheng University, Chiayi County, Taiwan; ^4^ Clinical Research Center, Ditmanson Medical Foundation Chia-Yi Christian Hospital, Chiayi City, Taiwan; ^5^ School of Nursing, China Medical University, Taichung, Taiwan; ^6^ Department of Nursing, China Medical University Hospital, Taichung, Taiwan; ^7^ School of Healthcare, China Medical University Hospital, Taichung, Taiwan

**Keywords:** head and neck cancer, turmeric, oral mucositis, radiotherapy, radiochemotherapy

## Abstract

**Background:**

Oral mucositis is a major complication for head and neck cancer (HNC) patients after radiotherapy or chemotherapy. A meta-analysis was performed to assess the efficacy of turmeric in the treatment of oral mucositis in HNC patients.

**Methods:**

Randomized controlled trials investigating our topic were included in the meta-analysis. The clinical outcomes considered were the severity of oral mucositis, pain level, and weight loss.

**Results:**

A total of eight articles that met our inclusion criteria were included in our meta-analysis. At the 3-week follow-up visit, the turmeric group showed significantly lower grades of oral mucositis compared to the control group (*p* = 0.03). When compared to the placebo group, a significant difference in the degree of oral mucositis was observed at the 4-(*p* = 0.03) and 6-week (*p* < 0.00001) follow-up visits. No significant difference in pain levels was observed between the turmeric and control groups at any of the follow-up visits. However, a significant improvement in pain levels for the turmeric group when compared with the placebo group was observed only at the 6-week follow-up visit (*p* = 0.006). Interestingly, a significant improvement in pain levels was observed for the turmeric group at the 2-, 4-, 5-, and 6-week follow-up visits (*p* < 0.05) when compared to the non-placebo group. The turmeric group showed less weight loss than the control group at the final follow-up visit (*p* = 0.03).

**conclusion:**

Our meta-analysis showed that using turmeric may be effective in improving both the severity of oral mucositis and pain levels in HNC patients who have received radiotherapy or radiochemotherapy. In addition, the turmeric group experienced less weight loss.

## Introduction

Head and neck cancer (HNC) was the seventh most commonly diagnosed cancer worldwide, with approximately 750,000 new cases reported and an estimated 370,000 deaths from the disease in 2020 according to the Globocan 2020 database (accessible online as part of the IARC Global Cancer Observatory). Patients with HNC may receive chemoradiotherapy for locally advanced cancer or surgical resection followed by adjuvant radiotherapy (Alfouzan, 2021). Oral mucositis is a major complication of radiotherapy, with 29%–66% of patients receiving a dose greater than 50 Gy experiencing severe symptoms (Lalla et al., 2008). Oral mucositis can cause severe pain in the throat, difficulty swallowing, and malnutrition, and it can also affect the quality of life and treatment compliance (Trotti et al., 2003; Sonis, 2009; Worthington et al., 2011). Therefore, it is crucial to search for an optimal therapy to improve oral mucositis in HNC patients after receiving radiotherapy.

Turmeric (*C*. *longa*) is a spice that is derived from the root of the Curcuma longa plant and is commonly used in traditional medicine. The three active ingredients in turmeric are curcumin, demethoxycurcumin, and bisdemethoxycurcumin. Curcumin is known for its anti-inflammatory and antioxidant properties (Gupta et al., 2012). Turmeric has been utilized to prevent and alleviate oral mucositis caused by chemotherapy and radiotherapy (Patil et al., 2015; Kia et al., 2021; Soni et al., 2022; Thomas et al., 2023). Otherwise, other therapies have been adopted to treat oral mucositis, such as chlorhexidine gluconate (Cardona et al., 2017) and zinc sulfate (Tian et al., 2018). Natural agents have shown promise in the treatment of chemotherapy-induced oral mucositis (Nagi et al., 2018). Therefore, further confirmation is needed to determine whether turmeric is a more optimal therapy for improving oral mucositis induced by chemotherapy and radiotherapy.

Meta-analysis is a statistical method used to pool the results of multiple reports on the same topic. These are considered the highest levels of evidence. A meta-analysis was previously conducted to analyze the efficacy of turmeric in improving oral mucositis in HNC patients who had received radiotherapy or radiochemotherapy (Dharman et al., 2021). Although they concluded that turmeric is safe and effective for oral mucositis in HNC patients, more detailed results have not been clarified by meta-analyses, such as the effect of turmeric at each follow-up visit, sensitive analysis, or possible sources of heterogeneity (Dharman et al., 2021). Therefore, further studies using meta-analysis should be conducted to investigate the efficacy of turmeric in the treatment of oral mucositis in HNC patients after radiotherapy or radiochemotherapy.

The objective of this study was to evaluate the effectiveness of turmeric in treating oral mucositis in HNC patients after radiotherapy or radiochemotherapy by using meta-analysis. The results could provide clinical information on the efficacy of turmeric in improving oral mucositis caused by radiotherapy or radiochemotherapy.

## Materials and methods

### Literature search

We followed the PRISMA (Preferred Reporting Items for Systematic Reviews and Meta-Analyses) statement to conduct this study (Page et al., 2021). The protocol was registered in the International Prospective Register of Systematic Reviews (PROSPERO), with the registration number CRD42023444339. Relevant articles were extensively searched in five electronic databases from their inception up to February 2024:PubMed, Cochrane Library, Embase, Web of Science, and ClinicalTrials. The keywords (“curcumin” OR “turmeric” OR “curcuma longa” OR “nanocurcumin“) AND “oral mucositis” OR “mucositis” were combined with their corresponding MeSH terms to search for potentially relevant articles across the five aforementioned databases. The detailed search process for each database is shown in Supplementary Table S1.

Two reviewers (CFW and HJW) independently searched for relevant articles. First, duplicate articles were identified and removed using EndNote software. Second, a preliminary review of titles and abstracts was conducted to identify potentially relevant articles. Finally, relevant articles were selected through full-text analysis. In cases of disagreements regarding the inclusion of literature, the two reviewers discussed the matter with the third author (T.P.Y) to reach a consensus.

### Inclusion and exclusion criteria

Articles were included in our study if they met the following criteria: 1) Patients with HNC were treated with chemotherapy or radiochemotherapy; 2) The experimental group consisted of patients who were treated with turmeric; 3) The control group consisted of patients who were treated with any type of therapy; 4) The study design was a randomized controlled trial; 5) the clinical outcomes included reported pain levels and the degree of oral mucositis. The exclusion criteria were as follows: 1) articles were review articles, conference abstracts, notes, and animal studies; 2) articles were not published in English or Chinese.

### Quality assessment

We used the updated Cochrane risk-of-bias tool for randomized trials (RoB 2) to evaluate the quality of each article included in the study (Sterne et al., 2019). This tool is used to evaluate the potential bias in each study based on five domains: 1) randomization process, 2) deviations from the intended intervention, 3) missing outcome data, 4) outcome measurement, and 5) selective reporting. Articles were classified as having a “high risk of bias,” “low risk of bias,” or “some concerns” based on the results of the aforementioned five domains. The two reviewers independently assessed the quality of each article, and any disagreements were resolved by discussion with the third author (T.R.Y) until a consensus was reached.

### Assessment of evidence

The certainty of the evidence for each outcome was assessed using the Grading of Recommendations, Assessment, Development, and Evaluation (GRADE) system (Guyatt et al., 2011). This method assigns a score of “high,” “moderate,” “low,” or “very low” based on the likelihood of bias, inconsistency, indirectness, imprecision, and other factors (like publication bias).

### Data extraction

We obtained the data by using a data extraction form that we developed ourselves. The data included the first author, year of publication, sample size, mean age, type of therapy, treatment course, and clinical outcomes. The two reviewers extracted the data independently, and any discrepancies were reviewed with the third author (T.R.Y) until a consensus was reached.

### Data analysis

All meta-analyses were performed using the Review Manager software (RevMan; version 5.4; The Nordic Cochrane Centre). The between-group treatment effect size of the included articles was pooled using a random effects model. The effect size was reported as standardized mean difference (SMD) and confidence interval (CI). A meta-analysis was performed for each follow-up visit. The value of *I* ^2^ was used to assess the statistical heterogeneity among the included articles. A value of *I* ^2^ between 30% and 60% indicated moderate heterogeneity, while a value greater than 60% indicated substantial or considerable heterogeneity. If heterogeneity was detected, the source of heterogeneity was further assessed. If the estimates extracted from the articles were zero, these values were replaced by 0.001 to conduct the meta-analysis.

## Results

### Literature search

**FIGURE 1:**
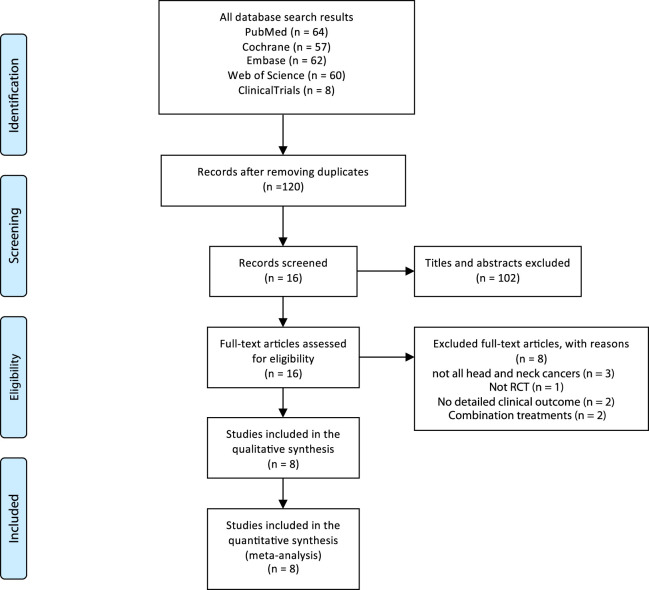
Flowchart of the search process for the five databases.

The literature search and selection process is depicted in Figure 1 using a flowchart. A total of 249 articles were initially identified in the five databases. After removing duplicates, 120 articles were screened for relevance based on their titles and abstracts. We retrieved and reviewed the full text of the remaining 16 articles to determine their eligibility. Of the total articles identified, 8 were excluded for various reasons: 1) not an RCT (n = 1); 2) no detailed clinical outcomes (n = 2); 3) combination treatments (n = 2); 4) not all patients with HNC (n = 3) (Supplementary Table S2). Finally, eight articles met our criteria for inclusion and were added to our meta-analysis (Rao et al., 2014; Patil et al., 2015; Charantimath, 2016; Delavarian et al., 2019; Arun et al., 2020; Shah et al., 2020; Kia et al., 2021; Soni et al., 2022; Thomas et al., 2023).

### Article characteristics

Table 1 shows the main characteristics of the included articles. The articles were published between 2014 and 2023. The majority of the eight studies were performed in India, with one performed in Iran. The articles had sample sizes ranging from 20 to 92, with a total of 503 participants. The age of the participants ranged from 40 to 62 years, with a gender distribution of 50%–90% male patients. The majority of the articles on oral mucositis assessment used World Health Organization (WHO) grading. One article used the Radiation Therapy Oncology Group (RTOG) scoring criteria, one used the National Cancer Institute Common Toxicity Criteria version 2 scale (NCI-CTC v.2), and one used the National Cancer Institute Common Terminology Criteria (NCICTC). In total, six articles assessed pain levels, with four using the numerical rating scale (NRS), one using the NCICTC, and one using the Patient Reported Oral Mucositis Symptom (PROMS). In total, four articles assessed weight loss after treatment.

**TABLE 1:** Main characteristics of the included articles.

First author, year, and country	Group type	Treatment	Cancer site	Cancer therapy	Main finding	Sample size n)	Mean age (year)	Sex (% of male patients)	Clinical outcome
Rao (2014), India	Experimental	Turmeric mouthwash	Tongue, buccal mucosa, retromolar trigone, palate, pharynx, alveolus, cheek, supraglottis, lip, pyriform sinus, tonsil, oral cavity, vallecula, or vocal cord	Radiotherapy or chemoradiotherapy	Turmeric gargling helped head and neck cancer patients who received radiation therapy by delaying and reducing the severity of mucositis	40	57	85	RTOG, weight lost
	Control	Povideone-iodine mouthwash				40	55	75	
Patil (2015), India	Experimental	Curcumin mouthwash	Oral cavity, pharynx, or larynx	Chemoradiotherapy	Curcumin outperformed chlorhexidine mouthwash in terms of faster wound healing and improved patient compliance in the treatment of oral mucositis caused by radiotherapy and chemotherapy	10	60	50	WHO, NRS
	Control	Chlorhexidine mouthwash				10	59	60	
Charantimath (2016), India	Experimental	Curcumin gel	NA	Chemoradiotherapy	Curcumin gel appeared to be an effective and safer option than chlorhexidine gel for the treatment of oral mucositis	20	NA	NA	WHO, NRS
	Control	Chlorhexidine gel				20	NA	NA	
Delavarian (2019), Iran	Experimental	Oral nanocurcumin	Buccal mucosa, tongue, or palate	Radiotherapy	Curcumin nanomicelle is a useful tool for preventing or reducing the severity of oral mucositis	16	62	56	NCI-CTC v2, weight lost
	Control	Placebo				16	56	63	
Arun (2020), India	Experimental	Oral turmeric extract	Oral cavity, oropharynx, glottis, or supraglottis	Radiotherapy or chemoradiotherapy	Patients receiving radiation therapy for head and neck cancer may benefit from the ability of turmeric extract to reduce the frequency and intensity of radiation-induced mucositis	30	NA	NA	WHO
	Control	Placebo				31	NA	NA	
Shah (2020), India	Experimental	Curcumin mouthwash	NA	Radiotherapy	While the use of 0.1% curcumin mouthwash was able to significantly delay the onset of RIOM, neither mouthwash was able to entirely prevent the onset of oral mucositis or reduce its severity	33	54	85	WHO, NRS
	Control	Benzydamine mouthwash				35	55	74	
Soni (2022), India	Experimental	Oral bio-enhanced turmeric formulation (low dose)	Oral cavity, buccal mucosa, hard palate, or floor of mouth	Chemoradiotherapy	Patients with oral cancer who experience severe oral mucositis, dysphagia, oral discomfort, and dermatitis due to chemotherapy and radiation therapy may greatly benefit from the bio-enhanced turmeric formulation	20	40	95	NCICTC for mucositis and pain, weight loss
	Experimental	Oral bio-enhanced turmeric formulation (high dose)				20	46	90	
	Control	Placebo				20	45	90	
Thomas (2023), India	Experimental	Turmeric mouthwash	Oral cavity, pharynx, or larynx	Radiotherapy or chemoradiotherapy	Compared to benzydamine mouthwash, turmeric mouthwash was more effective in reducing the severity of oral mucositis and related oral dysfunction	46	57	72	WHO, PROMS for pain, weight loss
	Control	Benzydamine mouthwash				46	58	78	

RTOG:Radiation Therapy Oncology Group; NCICTC:the National Cancer Institute Common Terminology Criteria; WHO: world health organization grading; PROMS: Patient Reported Oral Mucositis Symptom; NRS: numerical rating scale; NA: not available.

### Quality assessment

**FIGURE 2:**
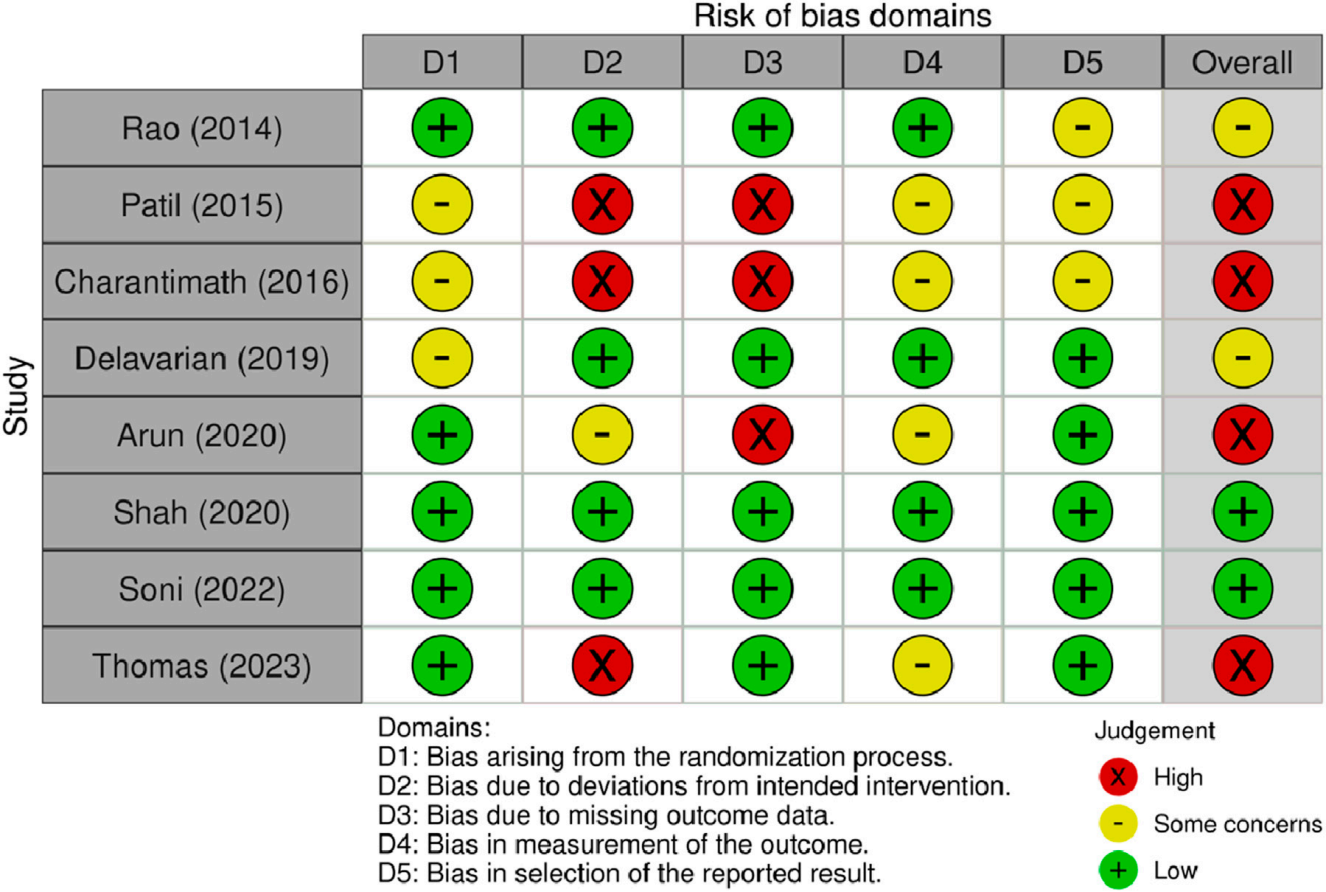
Summary of the risk of bias in the included articles according to the RoB2.0.

The quality assessment for each article is shown in Figure 2. For randomization bias, five articles had a low risk of bias, and the other three had some concerns about the methodology. Because of bias due to deviations from the intended intervention, three studies did not provide information on the blinding of participants and assessors and were graded as high risk. One article was graded as having some concerns due to single-blind participants. The other four articles had a low risk of bias. Because of bias due to missing outcome data, three articles did not report this information and were assessed as high risk. In total, four articles raised concerns about bias in outcome measures because the assessors may have been aware of which group the participants were in. Three articles were assessed with some concern for bias in the selection of reported results as they did not provide their protocols.

### Evidence assessment

Three clinical outcomes were used to evaluate the effectiveness of turmeric in oral mucositis: severity grade, pain level, and weight loss. Based on the GRADE system, the evidence for all three outcomes was determined to be moderate (Supplementary Table S3).

### Meta-analysis

### Overall effect size

Oral mucositis (severity grades)

**FIGURE 3:**
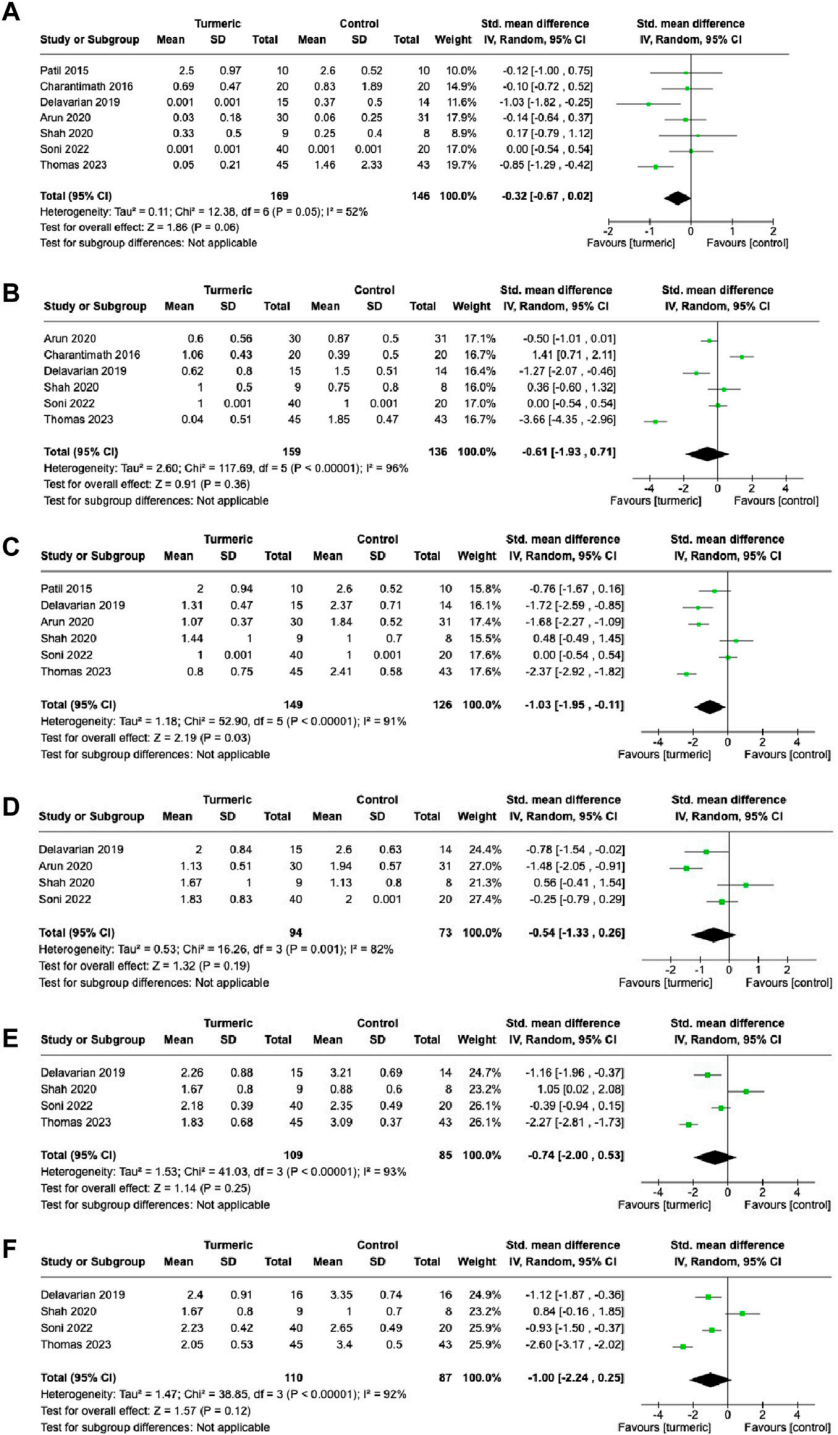
Comparison of the degree of oral mucositis between the experimental and control groups at 1-week **(A)**, 2-week **(B)**, 3-week **(C)**, 4-week **(D)**, 5-week **(E)**, and 6-week **(F)** follow-up visits.

The turmeric group showed no significant difference in the degree of oral mucositis compared with the control group at the 1- (SMD = −0.32, 95%CI = −0.67–0.02, *p* = 0.06, *I* ^2^ = 52%, n = 7) and 2-week (SMD = −0.61, 95%CI = −1.93–0.71, *p* = 0.36, *I* ^2^ = 96%, n = 6) follow-up visits (Figures 3A,B). At the 3-week follow-up visit, the turmeric group again showed significantly lower grades than the control group (SMD = −1.03, 95%CI = −1.95∼−0.11, *p* = 0.03, n = 6), and substantial heterogeneity was detected (*I* ^2^ = 91%) (Figure 3C). At the 4-week, 5-week, and 6-week follow-up visits, no significant differences in grades were observed between the turmeric and control groups (SMD = −0.54, 95%CI = −1.33–0.26, *p* = 0.19, n = 4; SMD = −0.74, 95%CI = −2.00–0.53, *p* = 0.25, n = 4; SMD = −1.00, 95%CI = −2.24–0.25, *p* = 0.12, n = 4, respectively), and substantial heterogeneity was detected (*I* ^2^ = 82%–93%) (Figure 3D–F).

Further investigation was conducted on the effect of the turmeric group by dividing the control group treatment into placebo and non-placebo groups. The turmeric group had a significantly lower grades of oral mucositis than the placebo group at the 4- (SMD = −0.83, 95%CI = −1.61∼−0.06, *p* = 0.03, *I* ^2^ = 79%, n = 3) and 6-week (SMD = −1.00, 95%CI = −1.45∼−0.55, *p* < 0.00001, *I* ^2^ = 0%, n = 2) follow-up visits (Supplementary Table S1). However, there were no significant differences in the grades of oral mucositis between the turmeric and non-placebo groups at the follow-up visits (Supplementary Table S4).

### Pain level

**FIGURE 4:**
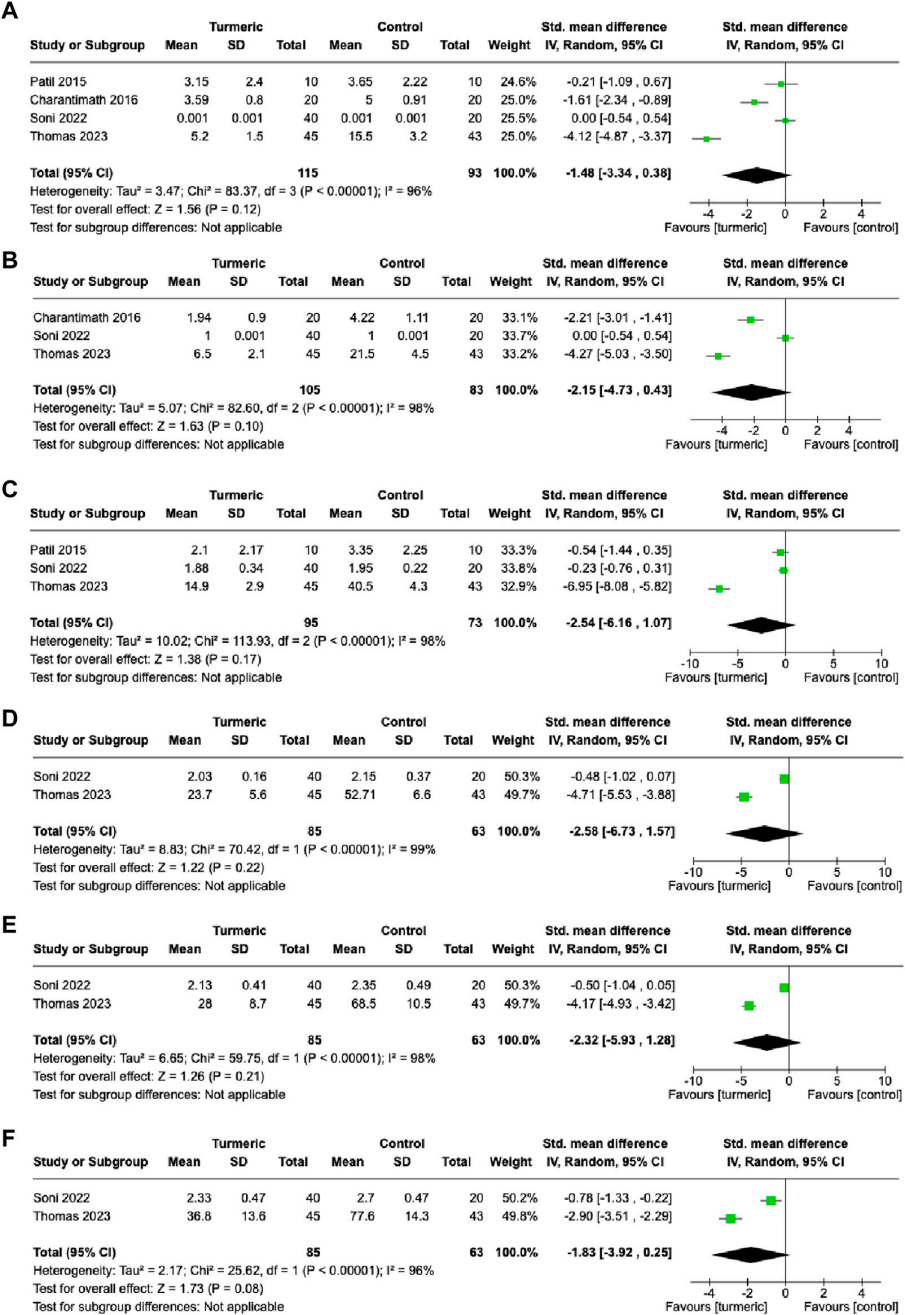
Comparison of pain levels between the experimental and control groups at 1-week **(A)**, 2-week **(B)**, 3-week **(C)**, 4-week **(D)**, 5-week **(E)**, and 6-week **(F)** follow-up visits.

At the 1-week follow-up visit, no significant difference was observed in pain levels between the turmeric and control groups (SMD = −1.48, 95%CI = −3.34–0.38, *p* = 0.12, n = 4), and substantial heterogeneity was detected (*I* ^2^ = 96%) (Figure 4A). Similarly, at the 2-, 3-, 4-, and 5-week follow-up visits, no significant difference in pain levels was observed between the turmeric and control groups (SMD = −2.15, 95%CI = −4.73–0.43, *p* = 0.10, n = 3; SMD = −2.54, 95%CI = −6.16–1.07, *p* = 0.17, n = 3; SMD = −2.58, 95%CI = −6.73–1.57, *p* = 0.22, n = 2; SMD = −2.32, 95%CI = −5.93–1.28, *p* = 0.21, n = 2; SMD = −1.83, 95%CI = −3.92–0.25, *p* = 0.08, n = 2, respectively), and substantial heterogeneity was detected (*I* ^2^ = 96–99%) (Figures 4B–F).

Further investigation was conducted on the effect of the turmeric group by dividing the control group treatment into placebo and non-placebo groups. The turmeric group had significantly lower pain scores than the placebo group at the 6-week follow-up (SMD = −0.78, 95%CI = −1.34∼−0.23, *p* = 0.006, n = 1) (Supplementary Table S5). The turmeric group had significantly lower pain score than the non-placebo group at the 2- (SMD = −3.24, 95%CI = −5.26∼−1.23, *I* ^2^ = 92%, *p* = 0.002, n = 2), 4-(SMD = −4.71, 95%CI = −5.53∼−3.88, *p* < 0.00001, n = 1), 5-(SMD = −4.17, 95%CI = −4.93∼−3.42, *p* < 0.00001, n = 1), and 6-week (SMD = −2.90, 95%CI = −3.51∼−2.29, *p* < 0.00001, n = 1) follow-up visits (Supplementary Table S5).

### Weight loss

**FIGURE 5:**
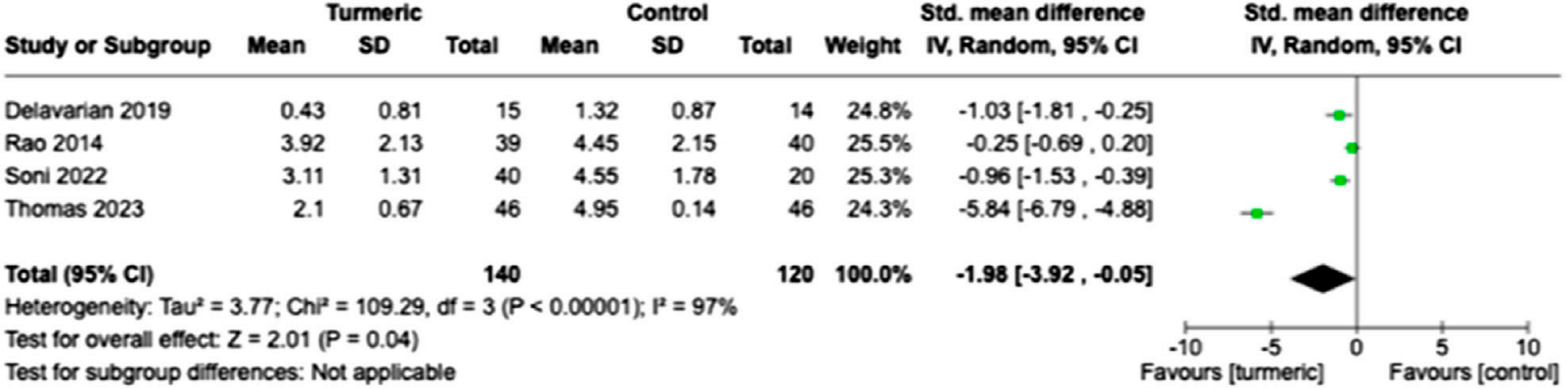
Comparison of weight loss between the experimental and control groups at the end of the follow-up period.

The weight loss of the participants was measured before and after treatment during the final follow-up period. The turmeric group showed significantly less weight loss than the control group (SMD = −1.98, 95% CI = −3.92 to −0.05, *p* = 0.04, n = 4). Substantial heterogeneity was detected (*I* ^2^ = 97%) (Figure 5).

### Sensitivity analysis

To conduct a sensitivity analysis, the meta-analysis was performed again by removing each article individually. After removing the articles by Shah et al. (2020) or Soni et al. (2022), the results changed and the turmeric group had significantly lower grades for oral mucositis than the control group at the 1-week follow-up visit (Table 2). At the 2-week follow-up visit, there were no changes in oral mucositis scores for sensitivity analysis. However, at the 3-week follow-up visit, the results changed. After removing the articles of Delavarian et al., 2019, Arun et al., 2020; Thomas 2023, no significant difference was observed. Similarly, at the 4-week follow-up visit, the result changed when the article by Shah et al. was removed. This pattern continued at the 5- and 6-week follow-up visits. With regard to pain levels, the group taking turmeric showed significantly lower pain levels at the 2-week follow-up visit, compared to the control group, after the article by Soni et al. was removed (Table 3). Regarding weight loss, there was no significant difference in weight loss between the two groups after the removal of the articles by Delavarian et al., Rao et al., or Soni et al. (Table 4).

**TABLE 2:** Sensitivity analysis for oral mucositis.

Subgroup (week)	Effect size				Study removed
	No. of articles	SMD	95%CI	*p*-value	
Oral mucositis					
1	6	−0.35	−0.73 to 0.04	0.08	Patil (2015)
1	6	−0.36	−0.76 to 0.03	0.07	Charantimath (2016)
1	6	−0.24	−0.58 to 0.10	0.17	Delavarian (2019)
1	6	−0.36	−0.77 to 0.05	0.08	Arun (2020)
1	6	−0.37	−0.74 to −0.01	0.04	Shah (2020)
1	6	−0.39	−0.77 to −0.01	<0.05	Soni (2022)
1	6	−0.18	−0.47 to 0.11	0.22	Thomas (2023)
2	5	−0.63	−2.37 to 1.10	0.47	Arun (2020)
2	5	−1.02	−2.36 to 0.32	0.14	Charantimath (2016)
2	5	−0.48	−2.03 to 1.07	0.54	Delavarian (2019)
2	5	−0.80	−2.30 to 0.71	0.30	Shah (2020)
2	5	−0.74	−2.41 to 0.93	0.39	Soni (2022)
2	5	−0.00	−0.81 to 0.81	0.99	Thomas (2023)
3	5	−1.08	−2.15 to −0.01	<0.05	Patil (2015)
3	5	−0.9	−1.97 to 0.17	0.10	Delavarian (2019)
3	5	−0.89	−2.03 to 0.25	0.12	Arun (2020)
3	5	−1.31	−2.25 to −0.37	0.007	Shah (2020)
3	5	−1.26	−2.25 to −0.38	0.005	Soni (2022)
3	5	−0.75	−1.63 to 0.13	0.09	Thomas (2023)
4	3	−0.44	−1.53 to 0.66	0.43	Delavarian (2019)
4	3	−0.22	−0.86 to 0.43	0.51	Arun (2020)
4	3	−0.83	−1.61 to −0.06	0.03	Shah (2020)
4	3	−0.63	−1.72 to 0.47	0.26	Soni (2022)
5	3	−0.58	−2.31 to 1.14	0.51	Delavarian (2019)
5	3	−1.28	−2.48 to −0.07	0.04	Shah (2020)
5	3	−0.84	−2.61 to 0.93	0.35	Soni (2022)
5	3	−0.22	−1.28 to 0.83	0.67	Thomas (2023)
6	3	−0.94	−2.66 to 0.78	0.28	Delavarian (2019)
6	3	−1.56	−2.65 to −0.46	0.005	Shah (2020)
6	3	−0.99	−2.85 to 0.86	0.29	Soni (2022)
6	3	−0.47	−1.49 to 0.55	0.36	Thomas (2023)

**TABLE 3:** Sensitivity analysis for pain level.

Subgroup (week)	Effect size				Removal of article
	No. of articles	SMD	95%CI	*p*-value	
Pain level					
1	3	−1.90	−4.28 to 0.48	0.12	Patil (2015)
1	3	−1.44	−4.28 to 1.19	0.28	Charantimath (2016)
1	3	−1.99	−4.18 to 0.21	0.08	Soni (2022)
1	3	−0.60	−1.63 to 0.43	0.25	Thomas (2023)
2	2	−2.12	−6.30 to 2.06	0.1	Charantimath (2016)
2	2	−3.24	−5.26 to −1.23	0.002	Soni (2022)
2	2	−1.08	−3.25 to 1.08	0.33	Thomas (2023)
3	2	−3.57	−10.16 to 3.02	0.29	Patil (2015)
3	2	−3.74	−10.02 to 2.54	0.24	Soni (2022)
3	2	−0.31	−0.77 to 0.15	0.19	Thomas (2023)

**TABLE 4:** Sensitivity analysis for weight loss.

OBS	Effect size				Removal of article
	No. articles	SMD	95%CI	*p*-value	
Weight loss					
1	3	−2.31	−4.92 to 0.30	0.08	Delavarian (2019)
2	3	−2.59	−5.35 to 0.18	0.07	Rao (2014)
3	3	−2.35	−5.36 to 0.66	0.13	Soni (2022)
4	3	−0.69	−1.23 to −0.15	0.01	Thomas (2023)

### Subgroup analysis

Substantial heterogeneity was found in almost all of the meta-analyses, indicating that some factors may influence the results. Therefore, subgroup analysis was conducted to determine the possible source of heterogeneity. The subgroup analysis was based on the type of treatment in the control group and the type of treatment in the turmeric group, respectively. Among the different types of control groups, the results demonstrated a significant difference in the grades of oral mucositis between the placebo and non-placebo groups at the 4-week follow-up visit (Supplementary Table S4). Similar results were also demonstrated for pain levels at the 2-, 4-, 5-, and 6-week follow-up visits (Supplementary Table S5). Regarding the different treatment types, a significant difference in the degree of oral mucositis between types was observed at the 2- and 4-week follow-up visits (Supplementary Table S6). Similar results in pain levels were also observed at the 1-, 2-, 4-, 5-, and 6-week follow-up visits (Supplementary Table S7).

## Discussion

Oral mucositis is a significant complication of radiotherapy that can cause severe pain in the throat, difficulty swallowing, malnutrition, and negatively impact quality of life and treatment compliance. This study aimed to evaluate the effectiveness of turmeric in the treatment of oral mucositis in HNC patients after radiotherapy or radiochemotherapy using meta-analysis. Our results show that turmeric could significantly reduce the degree of oral mucositis compared with a control group at the 3-week follow-up visit but no significant difference was observed in pain levels between the turmeric and control groups. Sensitivity analysis revealed that the results changed significantly when certain articles were removed. Moreover, statistical heterogeneity among the studies was found for most of the conducted meta-analyses. The heterogeneity was due to the type of control group and treatment methods. Furthermore, it was unclear whether there was publication bias among the included articles, as it is not optimal to assess bias with a small number of articles (n < 10) (Page et al., 2022).

The severity of oral mucositis is assessed using grades that increase with its severity. In a previous meta-analysis, the grade of oral mucositis was considered a categorical variable, and patients were considered to have an event if oral mucositis fell below a specific grade (Dharman et al., 2021). In our meta-analysis, we treated the grades as a continuous variable to compare the difference between the experimental and control groups. In the subgroup analysis, we found that the turmeric group had significantly lower grades at the 4-week and 6-week follow-up visits when compared with the placebo group, but not when compared with the non-placebo group (Supplementary Table S4). The non-placebo groups included povidone-iodine mouthwash, chlorhexidine mouthwash, chlorhexidine gel, and benzydamine mouthwash, which were used to treat oral mucositis in non-hematological cancer patients and had an effect on oral mucositis. Thus, the turmeric group should have a smaller difference in grades when compared with the non-placebo group than when compared with the placebo group. These results meet our expectations, considering the degree of oral mucositis as a continuous variable for performing meta-analyses.

In addition, the previous meta-analysis did not consider the impact of different follow-up visits on efficacy (Dharman et al., 2021; Zhang et al., 2021). The effectiveness of the intervention may vary at different follow-up visits, and this factor was confirmed in our meta-analysis. A previous report showed that the effect of curcumin on oral mucositis can be observed at the 3-week follow-up in HNC patients after radiotherapy, in comparison to the placebo group (Ramezani et al., 2023). Moreover, the type of control group (placebo or non-placebo) may also affect the efficacy. Different follow-up visits and types of control groups can influence the results of a meta-analysis. However, these factors were not considered in previous meta-analyses. These control groups can be classified into two types: placebo and non-placebo. The non-placebo group included different treatments, such as povidone-iodine mouthwash, chlorhexidine mouthwash, chlorhexidine gel, and benzylamine mouthwash. Povidone-iodine and benzylamine have been shown to be effective for oral mucositis in patients who received radiation therapy or chemotherapy (Kazemian et al., 2009; Kanagalingam et al., 2017). In the subgroup analysis, a significant difference in the degree of oral mucositis was observed between the two types of controls only at the 4-week follow-up visit (Supplementary Table S4). In addition, we found that the severity of oral mucositis was significantly reduced in the turmeric group compared to the placebo group at the 4- and 6-week follow-up visits. However, this difference was not observed when compared to the non-placebo group(Supplementary Table S4). These results indicate that the outcomes of the meta-analysis were influenced by differences in follow-up visits and the type of control used.

One of the effects of oral mucositis is weight loss, which increases the likelihood of a poor prognosis for cancer patients (Blakaj et al., 2019; Daly et al., 2020). The two previous meta-analyses showed that the turmeric group experienced less weight loss compared to the control group (Dharman et al., 2021; Zhang et al., 2021). These meta-analyses, however, only included two articles related to weight loss. In our meta-analysis, we included four articles to evaluate the difference in weight loss between the turmeric and control groups. Although our results showed that the turmeric group experienced less weight loss compared to the control group, the findings changed when we performed a sensitivity analysis. As we mentioned before, certain control groups were effective for oral mucositis. We found that the turmeric group experienced less weight loss when compared with the placebo group (SMD = −0.98, 95%CI = −1.44 to −0.53, *p* < 0.001, *I* ^2^ = 0%, n = 2). Our results demonstrated that the turmeric group was able to reduce weight loss after radiotherapy or chemoradiotherapy.

Sensitivity analysis is a crucial tool in meta-analyses to evaluate the consistency and dependability of the conclusions derived from the integration of multiple studies. A previous meta-analysis performed a sensitivity analysis for oral mucositis, and the results showed that no single article affected the overall effect (Zhang et al., 2021). However, they did not consider the impact of different follow-up visits on oral mucositis. In our study, we considered this impact, and the results were significantly influenced by a single article (Table 2). It would be reasonable to expect that the included articles contained different types of control groups. This variation in control groups should have an impact on the overall effect.

Pain is a common symptom of oral mucositis. Although a previous meta-analysis examined the difference in pain levels between the turmeric and control groups, only two articles were included in the analysis (Dharman et al., 2021). Moreover, different follow-up visits were subgrouped to investigate this issue. No significant difference in pain levels was observed at any of the follow-up visits (Figure 4). However, when different types of control groups were considered, a significant difference in pain levels between the two groups was observed at some follow-up visits. Interestingly, the turmeric group demonstrated a lower pain level at some follow-up visits when compared with the non-placebo group (Supplementary Table S5). Pain should be more sensitive than the oral mucositis grade in response to therapy. This indicates that the efficacy of turmeric is superior to that of other treatments. However, the current meta-analysis only included a few articles, so the conclusion may not be reliable.

Various treatment types of turmeric have been designed to improve oral mucositis, and they include mouthwash, gel, and other oral treatments (Charantimath, 2016; Shah et al., 2020; Kia et al., 2021; Soni et al., 2022; Thomas et al., 2023). However, the previous meta-analyses did not consider the differences between various treatment types (Dharman et al., 2021; Zhang et al., 2021). In our analysis, significant differences in the degree of oral mucositis were observed among the three treatment types at the 2- and 4- follow-up visits (Supplementary Table S6). Furthermore, we found that the oral treatment group experienced significant improvements in the degree of oral mucositis at the 4-, 5-, and 6-week follow-up visits compared to the control group. However, the oral treatment control groups were all given a placebo. Therefore, the oral treatment type was more likely to show significant improvement in the severity of oral mucositis. Significant differences in pain levels among the three treatment types were observed at the 1-, 2-, 4-, 5-, and 6-week follow-up visits (Supplementary Table S7). The mouthwash treatment appeared to have the greatest improvement in pain levels compared with the control group (Supplementary Table S7). However, the number of studies in the subgroup analysis was small (n ≤ 2). Thus, the results should be further confirmed in future studies.

In this study, we conducted a meta-analysis to evaluate the effectiveness of turmeric in the treatment of oral mucositis in HNC patients after radiotherapy or radiochemotherapy. We considered several factors in the meta-analysis, and our results provided more comprehensive findings compared to previous studies. Our findings suggest that turmeric is an effective and safe therapy for oral mucositis in HNC patients after radiotherapy or radiochemotherapy. It can be considered a replacement for current treatments. However, our study still has some limitations. First, although we included studies with a high level of evidence that investigated the effect of turmeric on oral mucositis in HNC patients after they received radiotherapy or radiochemotherapy, these articles used different treatment types and different control groups. These differences resulted in statistical heterogeneity in most of the meta-analyses, and a consistent result could not be obtained. Second, we performed a subgroup analysis to investigate the possible sources of statistical heterogeneity. Although different types of control groups and different treatment types significantly affected the results, a small number of articles (n < 3) in each subgroup could not reach convincing results. More studies should be conducted to investigate this topic in the future. Finally, these patients had different types of cancer and received different doses of radiation. These differences may affect our meta-analyses but could not be further investigated in this study.

In conclusion, this study investigated the effect of turmeric on oral mucositis in HNC patients who had undergone radiotherapy or radiochemotherapy. Our results demonstrated that turmeric improved oral mucositis in HNC patients compared to the placebo group. Moreover, turmeric appeared to be more effective in the pain relief than other treatments. Furthermore, the turmeric group experienced less weight loss than the placebo group. These results indicate that turmeric may be effective for HNC patients after radiotherapy or radiochemotherapy. The evidence in these results had moderate certainty. However, different types of turmeric treatment have been used to improve oral mucositis, and the best option could not be confirmed in this study. More studies should be conducted in the future to compare the efficacy of different types of turmeric treatment for oral mucositis in HNC patients after radiotherapy or radiochemotherapy.

## Data Availability

The original contributions presented in the study are included in the article/Supplementary Material, further inquiries can be directed to the corresponding authors.

## References

[B1] Alfouzan A. F. (2021). Radiation therapy in head and neck cancer. Saudi Med. J. 42, 247–254. 10.15537/smj.2021.42.3.20210660 33632902 PMC7989258

[B2] Arun P. Sagayaraj A. Azeem Mohiyuddin S. M. Santosh D. (2020). Role of turmeric extract in minimising mucositis in patients receiving radiotherapy for head and neck squamous cell cancer: a randomised, placebo-controlled trial. J. Laryngol. Otol. 134, 159–164. 10.1017/S0022215120000316 32029014

[B3] Blakaj A. Bonomi M. Gamez M. E. Blakaj D. M. (2019). Oral mucositis in head and neck cancer: evidence-based management and review of clinical trial data. Oral Oncol. 95, 29–34. 10.1016/j.oraloncology.2019.05.013 31345391

[B4] Cardona A. Balouch A. Abdul M. M. Sedghizadeh P. P. Enciso R. (2017). Efficacy of chlorhexidine for the prevention and treatment of oral mucositis in cancer patients: a systematic review with meta-analyses. J. Oral Pathol. Med. 46, 680–688. 10.1111/jop.12549 28075506

[B5] Charantimath S. (2016). Use of curcumin in radiochemotherapy induced oral mucositis patients: a control trial study. Int. Sch. Sci. Res. Innovation 10, 147–152.

[B6] Daly L. Dolan R. Power D. Ní Bhuachalla É. Sim W. Fallon M. (2020). The relationship between the BMI-adjusted weight loss grading system and quality of life in patients with incurable cancer. J. Cachexia Sarcopenia Muscle 11, 160–168. 10.1002/jcsm.12499 31692296 PMC7015235

[B7] Delavarian Z. Pakfetrat A. Ghazi A. Jaafari M. R. Homaei Shandiz F. Dalirsani Z. (2019). Oral administration of nanomicelle curcumin in the prevention of radiotherapy-induced mucositis in head and neck cancers. Spec. Care Dent. 39, 166–172. 10.1111/scd.12358 30761565

[B8] Dharman S. Shanmugasundaram K. Sampath R. K. (2021). A systematic review and meta-analysis on the efficacy of curcumin/turmeric for the prevention and amelioration of radiotherapy/radiochemotherapy induced oral mucositis in head and neck cancer patients. Asian Pac J. Cancer Prev. 22, 1671–1684. 10.31557/APJCP.2021.22.6.1671 34181321 PMC8418840

[B9] Gupta S. C. Patchva S. Koh W. Aggarwal B. B. (2012). Discovery of curcumin, a component of golden spice, and its miraculous biological activities. Clin. Exp. Pharmacol. Physiol. 39, 283–299. 10.1111/j.1440-1681.2011.05648.x 22118895 PMC3288651

[B10] Guyatt G. H. Oxman A. D. Schünemann H. J. Tugwell P. Knottnerus A. (2011). GRADE guidelines: a new series of articles in the Journal of Clinical Epidemiology. J. Clin. Epidemiol. 64, 380–382. 10.1016/j.jclinepi.2010.09.011 21185693

[B11] Kanagalingam J. Chopra A. Hong M. H. Ibrahim W. Villalon A. Lin J. C. (2017). Povidone-iodine for the management of oral mucositis during cancer therapy. Oncol. Rev. 11, 341. 10.4081/oncol.2017.341 28959380 PMC5607850

[B12] Kazemian A. Kamian S. Aghili M. Hashemi F. A. Haddad P. (2009). Benzydamine for prophylaxis of radiation-induced oral mucositis in head and neck cancers: a double-blind placebo-controlled randomized clinical trial. Eur. J. Cancer Care (Engl) 18, 174–178. 10.1111/j.1365-2354.2008.00943.x 19267733

[B13] Kia S. J. Basirat M. Saedi H. S. Arab S. A. (2021). Effects of nanomicelle curcumin capsules on prevention and treatment of oral mucosits in patients under chemotherapy with or without head and neck radiotherapy: a randomized clinical trial. BMC Complement. Med. Ther. 21, 232. 10.1186/s12906-021-03400-4 34521398 PMC8442420

[B14] Lalla R. V. Sonis S. T. Peterson D. E. (2008). Management of oral mucositis in patients who have cancer. Dent. Clin. North Am. 52, 61-77, viii, 10.1016/j.cden.2007.10.002 18154865 PMC2266835

[B15] Nagi R. Patil D. J. Rakesh N. Jain S. Sahu S. (2018). Natural agents in the management of oral mucositis in cancer patients-systematic review. J. Oral Biol. Craniofac Res. 8, 245–254. 10.1016/j.jobcr.2017.12.003 30191118 PMC6107930

[B16] Page M. J. Higgins J. P. Sterne J. A. (2022). Cochrane handbook for systematic reviews of interventions. chapter 13: assessing risk of bias due to missing results in a synthesis. https://training.cochrane.org/handbook/current/chapter-13.

[B17] Page M. J. Mckenzie J. E. Bossuyt P. M. Boutron I. Hoffmann T. C. Mulrow C. D. (2021). The PRISMA 2020 statement: an updated guideline for reporting systematic reviews. Syst. Rev. 10, 89. 10.1186/s13643-021-01626-4 33781348 PMC8008539

[B18] Patil K. Guledgud M. V. Kulkarni P. K. Keshari D. Tayal S. (2015). Use of curcumin mouthrinse in radio-chemotherapy induced oral mucositis patients: a pilot study. J. Clin. Diagn Res. 9, Zc59–62. 10.7860/JCDR/2015/13034.6345 PMC4576643 26436049

[B19] Ramezani V. Ghadirian S. Shabani M. Boroumand M. A. Daneshvar R. Saghafi F. (2023). Efficacy of curcumin for amelioration of radiotherapy-induced oral mucositis: a preliminary randomized controlled clinical trial. BMC Cancer 23, 354. 10.1186/s12885-023-10730-8 37069504 PMC10108802

[B20] Rao S. Dinkar C. Vaishnav L. K. Rao P. Rai M. P. Fayad R. (2014). The Indian spice turmeric delays and mitigates radiation-induced oral mucositis in patients undergoing treatment for head and neck cancer: an investigational study. Integr. Cancer Ther. 13, 201–210. 10.1177/1534735413503549 24165896

[B21] Shah S. Rath H. Sharma G. Senapati S. N. Mishra E. (2020). Effectiveness of curcumin mouthwash on radiation-induced oral mucositis among head and neck cancer patients: a triple-blind, pilot randomised controlled trial. Indian J. Dent. Res. 31, 718–727. 10.4103/ijdr.IJDR_822_18 33433509

[B22] Soni T. P. Gupta A. K. Sharma L. M. Singhal H. Sharma S. Gothwal R. S. (2022). A randomized, placebo-controlled study to evaluate the effect of bio-enhanced turmeric formulation on radiation-induced oral mucositis. ORL J. Otorhinolaryngol. Relat. Spec. 84, 103–113. 10.1159/000516577 34161952

[B23] Sonis S. T. (2009). Mucositis: the impact, biology and therapeutic opportunities of oral mucositis. Oral Oncol. 45, 1015–1020. 10.1016/j.oraloncology.2009.08.006 19828360

[B24] Sterne J. a.C. Savović J. Page M. J. Elbers R. G. Blencowe N. S. Boutron I. (2019). RoB 2: a revised tool for assessing risk of bias in randomised trials. BMJ 366, l4898. 10.1136/bmj.l4898 31462531

[B25] Thomas P. L. Kang H. K. Rishi K. S. (2023). Randomized control study of the effects of turmeric mouthwash on oral Health status, treatment-induced mucositis, and associated oral dysfunctions among patients with head and neck cancer. Cancer Nurs. 46, 36–44. 10.1097/NCC.0000000000001149 36066336

[B26] Tian X. Liu X. L. Pi Y. P. Chen H. Chen W. Q. (2018). Oral zinc sulfate for prevention and treatment of chemotherapy-induced oral mucositis: a meta-analysis of five randomized controlled trials. Front. Oncol. 8, 484. 10.3389/fonc.2018.00484 30510915 PMC6252385

[B27] Trotti A. Bellm L. A. Epstein J. B. Frame D. Fuchs H. J. Gwede C. K. (2003). Mucositis incidence, severity and associated outcomes in patients with head and neck cancer receiving radiotherapy with or without chemotherapy: a systematic literature review. Radiother. Oncol. 66, 253–262. 10.1016/s0167-8140(02)00404-8 12742264

[B28] Worthington H. V. Clarkson J. E. Bryan G. Furness S. Glenny A. M. Littlewood A. (2011). Interventions for preventing oral mucositis for patients with cancer receiving treatment. Cochrane Database Syst. Rev. 2011, Cd000978. 10.1002/14651858.CD000978.pub2 21491378 PMC7032547

[B29] Zhang L. Tang G. Wei Z. (2021). Prophylactic and therapeutic effects of curcumin on treatment-induced oral mucositis in patients with head and neck cancer: a meta-analysis of randomized controlled trials. Nutr. Cancer 73, 740–749. 10.1080/01635581.2020.1776884 32515617

