# Supplementary material for: Efficacy of turmeric in the treatment of oral mucositis in patients with head and neck cancer after radiotherapy or chemoradiotherapy: a systematic review and meta-analysis
Source: Front Pharmacol. 2024 Mar 15;15:1363202. doi: 10.3389/fphar.2024.1363202 (PMC10978772; doi:10.3389/fphar.2024.1363202)
Supplement: Supplementary file 1 [file Table1.DOCX]

Table S1. The detailed search process for each database.

| PubMed | |
| --- | --- |
| **10** | #6 AND #9 |
| **9** | #7 OR #8 |
| **8** | Oral Mucositis[MeSH Terms] |
| **7** | oral mucositis [Title/Abstract] |
| **6** | #1 OR #2 OR #3 OR #4 OR #5 |
| **5** | Curcumin[MeSH Terms] |
| **4** | nanocurcumin [Title/Abstract] |
| **3** | curcuma longa [Title/Abstract] |
| **2** | turmeric [Title/Abstract] |
| **1** | curcumin [Title/Abstract] |
| Cochrane Library | |
| **1** | (curcumin):ti,ab,kw OR (turmeric):ti,ab,kw OR (curcuma longa):ti,ab,kw OR (nanocurcumin):ti,ab,kw |
| **2** | MeSH descriptor: [Curcumin] explode all trees |
| **3** | #1 OR #2 |
| **4** | (oral mucositis):ti,ab,kw |
| **5** | MeSH descriptor: [Stomatitis] explode all trees |
| **6** | #4 OR #5 |
| **7** | #3 AND #6 |
| Embase | |
| **1** | (curcumin:ti,ab,kw OR turmeric:ti,ab,kw OR 'curcuma longa':ti,ab,kw OR nanocurcumin:ti,ab,kw) AND ('oral mucositis':ti,ab,kw OR mucositis:ti,ab,kw) |
| Web of science | |
| **1** | TS=(curcumin OR turmeric OR 'curcuma longa' OR nanocurcumin) |
| **2** | TS= ('oral mucositis' OR mucositis) |
| **3** | #1 AND #3 |
| ClinicalTrials | |
| **1** | Condition/ diseases: 'oral mucositis' OR mucositis |
| **2** | Intervention/treatment: curcumin OR turmeric OR 'curcuma longa' OR nanocurcumin |

Table S2. The detailed information of the excluded references.

| OBS | References | Exclusion reason |
| --- | --- | --- |
| 1 | F. Fardad, K. Ghasemi, N. Ansarinejad, N. Khodakarim, S. Nasiripour and M. Farasatinasab. A comparative study to assess the effectiveness of curcumin, mucosamin, and chlorhexidine in chemotherapy-induced oral mucositis. Explore 2023 Vol. 19 Issue 1 Pages 65-70. | Patients are not all head and neck cancer |
| 2 | M. Francis and S. Williams. Effectiveness of Indian Turmeric Powder with Honey as Complementary Therapy on Oral Mucositis: A Nursing Perspective among Cancer Patients in Mysore. The Nursing journal of India 2014 Vol. 105 Issue 6 Pages 258-260. | Not RCT |
| 3 | Kia, S. J., Basirat, M., Saedi, H. S., & Arab, S. A. (2021). Effects of nanomicelle curcumin capsules on prevention and treatment of oral mucosits in patients under chemotherapy with or without head and neck radiotherapy: a randomized clinical trial. BMC complementary medicine and therapies, 21(1), 232. | Patients are not all head and neck cancer |
| 4 | Mansourian , M. Amanlou , Sh. Shirazian, Z. Moosavian Jahromi , A. Amirian.(2015) The effect of “Curcuma Longa” topical gel on radiation -induced oral mucositis in patients with head and neck cancer. International Journal of Radiation Research,13(3), 269-274 | No detailed clinical outcome |
| 5 | A. F. L. Martins, C. H. Pereira, M. O. Morais, S. S. de Sousa-Neto, M. C. Valadares, N. M. A. Freitas, et al. Effects of a mucoadhesive phytomedicine (Curcuma longa L. and Bidens pilosa L.) on radiotherapy-induced oral mucositis and quality of life of patients undergoing head and neck cancer treatment: randomized clinical trial. Supportive Care in Cancer 2023 Vol. 31 Issue 9. | Combination treatments |
| 6 | V. Ramezani, S. Ghadirian, M. Shabani, M. A. Boroumand, R. Daneshvar and F. Saghafi. Efficacy of curcumin for amelioration of radiotherapy-induced oral mucositis: a preliminary randomized controlled clinical trial. BMC Cancer 2023 Vol. 23 Issue 1. | No detailed clinical outcome |
| 7 | S. Revel-Vilk, Y. Zadik, I. Meidan, G. Salam, S. Simaan, I. Zeevi, et al. Topical curcumin for the prevention of oral mucositis in pediatric patients. Supportive Care in Cancer 2012 Vol. 20 Pages S183. | Patients are not all head and neck cancer |
| 8 | E. X. D. Santos Filho, D. A. C. Arantes, A. F. Oton Leite, A. C. Batista, E. F. Mendonça, R. N. Marreto, et al. Randomized clinical trial of a mucoadhesive formulation containing curcuminoids (Zingiberaceae) and Bidens pilosa Linn (Asteraceae) extract (FITOPROT) for prevention and treatment of oral mucositis - phase I study. Chem Biol Interact 2018 Vol. 291 Pages 228-236. | Combination treatments |

RCT: randomized controlled trial.

Table S3. The certainty of evidence for each outcome assessed using GRADE.

|  | | | | | | | | | | | |
| --- | --- | --- | --- | --- | --- | --- | --- | --- | --- | --- | --- |
| **Certainty assessment** | | | | | | | **Summary of findings** | | | | |
| **Participants (studies) Follow-up** | **Risk of bias** | **Inconsistency** | **Indirectness** | **Imprecision** | **Publication bias** | **Overall certainty of evidence** | **Study event rates (%)** | | **Relative effect (95% CI)** | **Anticipated absolute effects** | |
|  |  |  |  |  |  |  | **With [comparison]** | **With Turmeric** |  | **Risk with [comparison]** | **Risk difference with Turmeric** |
| **Oral mucositis (follow-up: range 3 weeks to 6 weeks; assessed with: O; Scale from: 0 to 5)** | | | | | | | | | | | |
| 275 (6 RCTs) | serious | serious^a^ | not serious | serious^b^ | all plausible residual confounding would reduce the demonstrated effect dose response gradient | ⨁⨁⨁◯ Moderate | 126 | 149 | - | - | SMD **1.03 SD lower** (1.95 lower to 0.11 lower) |
| **Pain level (follow-up: range 3 weeks to 6 weeks; Scale from: 0 to 10)** | | | | | | | | | | | |
| 188 (3 RCTs) | serious^a^ | serious | not serious | serious | all plausible residual confounding would reduce the demonstrated effect dose response gradient | ⨁⨁⨁◯ Moderate | 83 | 105 | - | - | SMD **2.54 SD fewer** (6.16 fewer to 1.07 more) |
| **weight loss (follow-up: range 3 weeks to 6 weeks; Scale from: 0 to 70)** | | | | | | | | | | | |
| 260 (4 RCTs) | serious | serious | not serious | serious | all plausible residual confounding would reduce the demonstrated effect dose response gradient | ⨁⨁⨁◯ Moderate | 120 | 140 | - | - | SMD **1.98 SD lower** (3.92 lower to 0.05 lower) |

**CI:** confidence interval; **SMD:** standardized mean difference

#### Explanations

a. After subgroup analysis, heterogeneity is still high.

b. The sample size is too small

Table S4. Subgroup analysis for oral mucositis based on different control types.

| Control group | Effect size | | | |  | Heterogeneity | Subgroup-differences |
| --- | --- | --- | --- | --- | --- | --- | --- |
|  | No. Studies | SMD | 95%CI | P-value |  | *I*^2^ | P-value |
| ***1-week*** |  |  |  |  |  |  |  |
| Placebo | 3 | -0.32 | -0.85 to 0.22 | 0.25 |  | 59 | 0.99 |
| no-placebo | 4 | -0.32 | -0.83 to 0.19 | 0.22 |  | 55 |  |
| ***2-week*** |  |  |  |  |  |  |  |
| Placebo | 3 | -0.53 | -1.17 to 0.11 | 0.10 |  | 70 | 0.95 |
| no-placebo | 3 | -0.63 | -3.93 to 2.66 | 0.71 |  | 98 |  |
| ***3-week*** |  |  |  |  |  |  |  |
| Placebo | 3 | -1.11 | -2.32 to 0.10 | 0.07 |  | 90 | 0.86 |
| no-placebo | 3 | -0.92 | -2.65 to 0.82 | 0.30 |  | 93 |  |
| ***4-week*** |  |  |  |  |  |  |  |
| Placebo | 3 | -0.83 | -1.61 to -0.06 | 0.03 |  | 79 | 0.03 |
| no-placebo | 1 | 0.56 | -0.41 to 1.54 | 0.26 |  | - |  |
| ***5-week*** |  |  |  |  |  |  |  |
| Placebo | 2 | -0.72 | -1.47 to 0.02 | 0.06 |  | 59 | 0.96 |
| no-placebo | 2 | -0.64 | -3.89 to 2.61 | 0.70 |  | 97 |  |
| ***6-week*** |  |  |  |  |  |  |  |
| Placebo | 2 | -1.00 | -1.45 to -0.55 | <0.00001 |  | 0 | 0.96 |
| no-placebo | 2 | -0.90 | -4.27 to 2.47 | 0.60 |  | 97 |  |

Table S5. Subgroup analysis for pain level based on different control types.

| Subgroup | Effect size | | | |  | Heterogeneity | Subgroup-differences |
| --- | --- | --- | --- | --- | --- | --- | --- |
|  | No. Studies | SMD | 95%CI | P-value |  | *I*^2^ | P-value |
| ***1-week*** |  |  |  |  |  |  |  |
| Placebo | 1 | 0.00 | -0.54 to 0.54 | 1.00 |  | - | 0.08 |
| no-placebo | 3 | -1.99 | -4.18 to 0.21 | 0.08 |  | 96 |  |
| ***2-week*** |  |  |  |  |  |  |  |
| Placebo | 1 | 0.00 | -0.54 to 0.54 | 1.00 |  | - | 0.002 |
| no-placebo | 2 | -3.24 | -5.26 to -1.23 | 0.002 |  | 92 |  |
| ***3-week*** |  |  |  |  |  |  |  |
| Placebo | 1 | -0.23 | -0.76 to 0.31 | 0.41 |  | - | 0.28 |
| no-placebo | 2 | -3.74 | -10.02 to 2.54 | 0.24 |  | 99 |  |
| ***4-week*** |  |  |  |  |  |  |  |
| Placebo | 1 | -0.48 | -1.02 to 0.07 | 0.09 |  | - | <0.00001 |
| no-placebo | 1 | -4.71 | -5.53 to -3.88 | <0.00001 |  | - |  |
| ***5-week*** |  |  |  |  |  |  |  |
| Placebo | 1 | -0.50 | -1.04 to 0.04 | 0.07 |  | - | <0.00001 |
| no-placebo | 1 | -4.17 | -4.93 to -3.42 | <0.00001 |  | - |  |
| ***6-week*** |  |  |  |  |  |  |  |
| Placebo | 1 | -0.78 | -1.34 to -0.23 | 0.006 |  | - | <0.00001 |
| no-placebo | 1 | -2.90 | -3.51 to -2.29 | < 0.00001 |  | - |  |

Table S6. Subgroup analysis for oral mucositis based on different treatment types.

| Treatment type | Effect size | | | |  | Heterogeneity | Subgroup-differences |
| --- | --- | --- | --- | --- | --- | --- | --- |
|  | No. Studies | SMD | 95%CI | P-value |  | *I*^2^ | P-value |
| ***1-week*** |  |  |  |  |  |  |  |
| Mouthwash | 3 | -0.38 | -1.05 to 0.29 | 0.27 |  | 59 | 0.81 |
| Gel | 1 | -0.10 | -0.72 to 0.52 | 0.75 |  | - |  |
| Oral | 3 | -0.32 | -0.85 to 0.22 | 0.25 |  | 59 |  |
| ***2-week*** |  |  |  |  |  |  |  |
| Mouthwash | 2 | -1.66 | -5.60 to 2.27 | 0.41 |  | 98 | 0.0002 |
| Gel | 1 | 1.41 | 0.71 to 2.11 | <0.0001 |  | - |  |
| Oral | 3 | -0.53 | -1.17 to 0.11 | 0.10 |  | 70 |  |
| ***3-week*** |  |  |  |  |  |  |  |
| Mouthwash | 3 | -0.92 | -2.65 to 0.82 | 0.30 |  | 93 | 0.86 |
| Gel | - | - | - | - |  | - |  |
| Oral | 3 | -1.11 | -2.32 to 0.10 | 0.08 |  | 90 |  |
| ***4-week*** |  |  |  |  |  |  |  |
| Mouthwash | 1 | 0.56 | -0.41 to 1.54 | 0.26 |  | - | 0.01 |
| Gel | - | - | - |  |  | - |  |
| Oral | 3 | -0.92 | -1.55 to -0.28 | 0.005 |  | 69 |  |
| ***5-week*** |  |  |  |  |  |  |  |
| Mouthwash | 2 | -0.64 | -3.89 to 2.61 | 0.70 |  | 97 | 0.94 |
| Gel | - | - | - | - |  | - |  |
| Oral | 2 | -0.76 | -1.40 to -0.12 | 0.02 |  | 45 |  |
| ***6-week*** |  |  |  |  |  |  |  |
| Mouthwash | 2 | -0.90 | -4.27 to 2.47 | 0.60 |  | 97 | 0.96 |
| Gel | - | - | - | - |  | - |  |
| Oral | 2 | -0.81 | -1.26 to -0.36 | 0.0005 |  | 0 |  |

Table S7. Subgroup analysis for pain level based on different control types.

| Subgroup | Effect size | | | |  | Heterogeneity | Subgroup-differences |
| --- | --- | --- | --- | --- | --- | --- | --- |
|  | No. Studies | SMD | 95%CI | P-value |  | *I*^2^ | P-value |
| ***1-week*** |  |  |  |  |  |  |  |
| Mouthwash | 2 | -2.17 | -6.00 to 1.66 | 0.27 |  | 98 | 0.002 |
| Gel | 1 | -1.61 | -2.34 to -0.89 | <0.0001 |  | - |  |
| Oral | 1 | 0.00 | -0.54 to 0.54 | 1.00 |  | - |  |
| ***2-week*** |  |  |  |  |  |  |  |
| Mouthwash | 1 | -4.27 | -5.03 to -3.50 | <0.00001 |  | - | <0.00001 |
| Gel | 1 | -2.21 | -3.01 to -1.41 | <0.00001 |  | - |  |
| Oral | 1 | 0.00 | -0.54 to 0.54 | 1.00 |  | - |  |
| ***3-week*** |  |  |  |  |  |  |  |
| Mouthwash | 2 | -3.74 | -10.02 to 2.54 | 0.24 |  | 99 | 0.28 |
| Gel | - | - | - | - |  | - |  |
| Oral | 1 | -0.23 | -0.76 to 0.31 | 0.41 |  | - |  |
| ***4-week*** |  |  |  |  |  |  |  |
| Mouthwash | 1 | -4.71 | -5.53 to -3.88 | <0.00001 |  | - | <0.00001 |
| Gel | - | - | - | - |  | - |  |
| Oral | 1 | -0.48 | -1.02 to 0.07 | 0.09 |  | - |  |
| ***5-week*** |  |  |  |  |  |  |  |
| Mouthwash | 1 | -4.17 | -4.93 to -3.42 | <0.00001 |  | - | <0.00001 |
| Gel | - | - | - | - |  | - |  |
| Oral | 1 | -1.02 | -1.59 to -0.45 | 0.0004 |  | - |  |
| ***6-week*** |  |  |  |  |  |  |  |
| Mouthwash | 1 | -2.90 | -3.51 to -2.29 | <0.00001 |  | - | <0.00001 |
| Gel | - | - | - | - |  | - |  |
| Oral | 1 | -0.78 | -1.34 to -0.23 | 0.006 |  | - |  |
